# The Academic Medical Center Linear Disability Score for evaluation of physical reserve on admission to the ICU: can we query the relatives?

**DOI:** 10.1186/cc10447

**Published:** 2011-09-14

**Authors:** José GM Hofhuis, Marcel GW Dijkgraaf, Aly Hovingh, Richard L Braam, Lisa van de Braak, Peter E Spronk, Johannes H Rommes

**Affiliations:** 1 Department of Intensive Care, Gelre Hospital, Apeldoorn, Albert Schweitzerlaan 31, 7334 DZ Apeldoorn, the Netherlands; 2 Clinical Research Unit, Academic Medical Center, Amsterdam, Meibergdreef 9, 1105 AZ Amsterdam, the Netherlands; 3 Department of Cardiology, Gelre Hospital, Apeldoorn, Albert Schweitzerlaan 31, 7334 DZ Apeldoorn, the Netherlands; 4 Department of Intensive Care, Academic Medical Center, Amsterdam, Meibergdreef 9, 1105 AZ Amsterdam, the Netherlands

## Abstract

**Introduction:**

Evaluating the pre-morbid functional status in critically ill patients is important and frequently done using the physical component score (PCS) of the Short Form 36, although this approach has its limitations. The Academic Medical Center Linear Disability Score (ALDS) is a recently developed generic item bank used to measure the disability status of patients with a broad range of diseases. We aimed to study whether proxy scoring with the ALDS could be used to assess the patients' functional status on admission for cardiac care unit (CCU) or ICU patients and how the ALDS relates to the PCS using the Short Form 12 (SF-12).

**Methods:**

Patients and proxies completed the ALDS and SF-12 score in the first 72 hours following ICU scheduled surgery (*n *= 14), ICU emergency admission (*n *= 56) and CCU emergency admission (*n *= 70).

**Results:**

In all patients (*n *= 140) a significant intra-class correlation was found for the ALDS (0.857), the PCS (0.798) and the mental component score (0.679) between patients and their proxy. In both scheduled and emergency admissions, a significant correlation was found between patients and their proxy for the ALDS, although the lowest correlation was found for the ICU scheduled admissions (0.755) compared with the ICU emergency admissions (0.889). In CCU patients, the highest significant correlation between patients and proxies was found for the ALDS (0.855), for the PCS (0.807) and for the mental component score (0.740).

**Conclusions:**

Relatives in close contact with critically ill patients can adequately reflect the patient's level of disability on ICU and CCU admission when using the ALDS item bank, which performed at least as well as the PCS. The ALDS could therefore be a useful alternative for the PCS of the SF-12.

## Introduction

To assess the effects of critical illness and treatment in the ICU on changes in disability status, measurements evaluating health-related quality of life (HRQOL) should be performed at ICU admission. As most patients are not able to complete questionnaires at the time of admission, proxies must frequently be used as a surrogate. Assessment of the functional status on admission provides valuable information that could support the physician in decision-making regarding ICU admission and withholding life-sustaining treatment in a critically ill patient. However, can proxies provide useful and reliable information on functional status in critically ill patients? Some studies in critically ill patients reported moderate agreement between individual patients and their proxies, although lower levels of agreement may be reported for psychological functioning [1,2]. In a previous study performed by our group, we found that proxies adequately assess the patients' quality of life on admission to the ICU when using the Short Form 36 (SF-36) [3]. The SF-36 is based on calculating sum-scores, however, and there are some disadvantages with the use of sum-scores such as the unclear clinical significance of the sum-scores and the difficulty to compare the results of repeated health measures [4].

The assessment of the functional health status on ICU admission is important to judge the biological reserve of a patient in relation to final outcome [5,6]. Current measures such as the physical component score (PCS) of the widely used SF-36, however, have several limitations; for instance, the SF-36 scores are used to calculate a summarised score with an inherent loss of information. Using a questionnaire based on item response theory may overcome these problems. The Academic Medical Center Linear Disability Score (ALDS) is a recently developed generic item bank that measures the disability status of patients with a broad range of diseases, as expressed by the ability to perform activities in daily living [7]. Using the ALDS, it is possible to arrange both the item difficulty and the patient's ability on a single hierarchical linear scale. This method makes it possible to present different sets of items to different groups of patients. Since all of the items are calibrated, the measurements remain comparable. Another advantage of the ALDS is its simplicity to use for both patients and their relatives directly after ICU admission. The ALDS has been validated in a large, mixed patient population [8], and in patients with rheumatoid arthritis [9] and with Parkinson's disease [10]. The ALDS has not, however, been validated for critically ill patients or for use by proxies to estimate ICU preadmission functional status.

The aims of our study were to evaluate whether proxy scoring with the ALDS and the Short Form 12 (SF-12) can be used to assess the patients' functional status on admission to the coronary care unit (CCU) or ICU and, secondly, to evaluate how the physical disability score of the ALDS relates to the PCS using the SF-12.

## Materials and methods

### Patients and proxies

Patients eligible for the study were those admitted to the ICU and CCU of the Gelre hospital, a 654-bed university-affiliated hospital with a 12-bed mixed ICU and a 10-bed CCU. Patients and proxies were asked to complete the ALDS and the SF-12 within 72 hours following ICU or CCU admission using interviews. Proxies had to be in close contact with the patient on a regular basis. Standard instructions were provided in which proxies were asked to try to view the patient's physical health in the last 4 weeks prior to admission, from the patient's perspective. Additionally, all items were made from the third-person perspective (for example, 'would the patient say that he/she ...'). Proxies completed the interviews in a separate room, when possible simultaneously with the patients. All interviews were taken by two skilled investigators. The study was approved by the local ethics committee of the Gelre Hospital location Apeldoorn, the Netherlands.

### The Academic Medical Center Linear Disability Score

The ALDS is a recently developed generic item bank that measures disability status, as expressed by the ability to perform activities of daily life [8]. In contrast to the widely used sum-score-based questionnaires, the ALDS item bank was developed within the framework of the item-response theory. The ALDS item bank covers a large number of activities, which are suitable for assessing respondents with a very wide range of functional status. Each item in the ALDS item bank describes an activity of daily life; examples include 'walking for more than 15 minutes', 'showering', and 'washing up'. The items were obtained from a systematic review of generic and disease-specific instruments designed to measure functional status [11]. Each item has two response categories: 'I could carry out the activity' and 'I could not carry out the activity'. If the patient had not been able to experience a specific activity, a 'not applicable' response was recorded.

The advantage of the ALDS is that an item bank is a collection of items for which the measurement properties of each item are known [12,13]. Since item response theory focuses on the measurement properties of individual items, rather than the instrument as a whole, it is not essential for all respondents to be examined using all items when using an item bank. It is possible to select items for individual patients using small sets of items tailored to the disability levels of the patients [14]. Questions are asked based on the answers of the patients. For instance, it is pointless to ask a patient 'Are you able to go for a walk in the woods?' if he previously stated not being able to go for a short walk of 15 minutes. This can reduce the burden of testing considerably for both patients and researchers, and can reduce the time needed to answer the required number of questions.

In the present study, the ALDS was applied as a computer adaptive test. In short, a patient's level of functional status was initially estimated based on his response pattern to five items that were randomly selected from the quintiles of the item bank (see Additional file 1 for the hierarchy of the ALDS items). Based on this initial estimate, the next item was selected at the targeted functional status level and the response to this item was used to adjust the initial estimate. This procedure continued until a prior specified minimum reliability of the final estimated was established. The computer adaptive test application will be commercially available by the end of 2011 (for further details, please contact MGWD). The ALDS can still be applied as a paper-and-pencil instrument, in which case the researcher *a priori *selects approximately 20 to 25 items from the item bank that are relevant for the whole target population at the time of measurement.

The original units of the ALDS scale are (logistic) regression coefficients, expressed in logits. For interpretation, the logit scores were linearly transformed into values between 0 (dead) and 100, with 1 representing the lowest and 100 the highest level of functional status possible [15]. The average time required to complete the ALDS item bank as well as the SF-12 in our study was 5 to 10 minutes.

### Short Form 12

The SF-12 health survey is a generic measure of health status that was developed to provide an alternative to the SF-36 for purposes of monitoring large samples from general and specific populations. The SF-12 items allow the allocation of the PCS and the mental component score (MCS) summary scores but not of the subscales of the original SF-36. The SF-12 is short, easy to administer and the items are a subset of those in the SF-36. The SF-12 has proven reliability and validity [16]. This subset was selected because it best reproduces the SF-36 PCS and MCS summary scores in general and specific populations and because the set represents all eight SF-36 dimensions [16]. Higher SF-12 scores indicate better HRQOL, a positive change in SF-12 scores indicates improvement of HRQOL and a negative change indicates deterioration [17].

### Statistical analyses

The intra-class correlation coefficients were used to estimate the absolute agreement levels between patients and proxies regarding the ALDS as well as the PCS and MCS of the SF-12. Estimated intra-class correlation coefficients were interpreted as follows: *≤*0.40, poor to fair agreement; 0.41 to 0.60, moderate agreement; 0.61 to 0.80, good agreement; 0.81 to 1.00, excellent agreement [18]. The intra-class correlation coefficient has been demonstrated to be mathematically equivalent to the weighted kappa statistic [19].

A Pearson's chi-square test was used to assess demographic differences between ICU scheduled admissions and ICU emergency admissions and between ICU patients and CCU patients. The level of agreement between patients and their proxies regarding the ALDS both in ICU and CCU patients was assessed with the Bland-Altman method and visually with a Bland-Altman plot [20]. A Mann-Whitney test was used to assess differences between proxies' gender, age and relationship with the patient. *P *< 0.05 was considered statistically significant. All data are expressed as the mean ± standard deviation or the median and interquartile range (P_25 _to P_75_). Data were analysed using the Statistical Package for the Social Sciences (version 13; SPSS Inc., Chicago, IL, USA).

## Results

**Figure 1:**
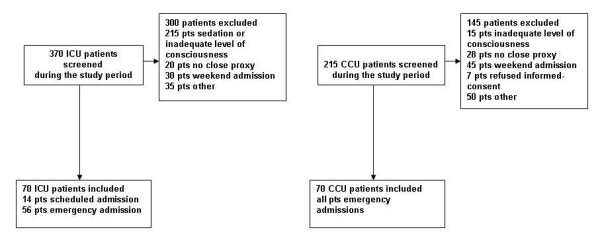
**Flow diagram of the patients screened and included in the study**. CCU, coronary care unit; pts, patients.

Between January 2010 and October 2010, 370 admissions were screened on the ICU. In total, 300 patients were excluded for a variety of reasons (Figure 1). Between January 2010 and June 2010, 215 admissions were screened on the CCU. A total of 145 patients were excluded (Figure 1). We included a total of 140 patients in the study (88 men, 52 women) for acute ICU admissions (*n *= 56), scheduled ICU admissions (*n *= 14) and acute CCU admissions (*n *= 70). Those patients and proxies completed the ALDS and the SF-12. Demographic and clinical characteristics of all study patients are shown in Table 1.

**Table 1:** Demographic and clinical characteristics of patients and proxies

	All patients	All ICU patients	ICU scheduled admissions	ICU emergency admissions	All CCU emergency admissions	*P *value^a^
*n*	140	70	14	56	70	14/56
Age (years)	73.5 (63 to 88)	70 (59 to 80)	67 (61 to 78)	70 (59 to 83)	78(70 to 83)	0.378
Sex, male/female (%)	63.2/36.8	66.4/33.6	64.3/35.7	66.4/33.6	60/40	0.832
APACHE II score	-	14 (11 to 16)	12 (10 to 16.2)	14 (12 to 16)	-	0.502
SAPS	-	29 (22 to 36)	22 (17 to 31)	30 (26 to 37)	-	0.333
ICU/CCU length of stay (days)	-	2 (2 to 4)	2 (2 to 3)	2 (2 to 4)	3 (2 to 4)	0.432
Hospital length of stay (days)	-	15.5 (9 to 24)	15 (10 to 26)	15 (8 to 23)	7 (4.7 to 11)	0.530
Ventilation days	-	1 (0 to 2)	1.5 (0.7 to 2)	0 (0 to 2)	-	0.075
Diagnostic groups						
Cardiovascular	85 (60.7)	15 (21.4)	2 (14.3)	15 (26.8)	70 (100)	0.431
Respiratory	11 (7.9)	11 (15.7)	3 (21.4)	8 (14.3)	-	0.581
Gastrointestinal	32 (22.9)	32 (45.7)	5 (35.7)	25 (44.6)	-	0.694
Neurological	1 (0.7)	1 (1.4)	-	-	-	-
Urological	6 (4.3)	6 (8.6)	4 (28.6)	3 (5.4)	-	0.026
Others	5 (3.6)	5 (7.1)	-	5 (8.9)	-	-
Type of proxy						
Spouse	96 (68.6)	47 (67.1)	11 (78.6)	36 (64.3)	48 (68.6)	0.659
Child	40 (28.6)	20 (28.6)	2 (14.3)	18 (32.1)	22 (31.4)	0.302
Brother/sister	2 (1.5)	1 (1.4)	1 (7.1)	-	-	-
Parents	2 (1.5)	2 (2.9)	-	2 (3.6)	-	-
						

Data presented as the median (interquartile range, P_25 _to P_75_) or *n *(%). APACHE II, Acute Physiology and Chronic Health Evaluation; CCU, coronary care unit; SAPS, Simplified Acute Physiology Score.^a^ICU scheduled admissions and ICU emergency admissions.

### Agreement between proxy and patient responses

#### All patients

**Figure 2:**
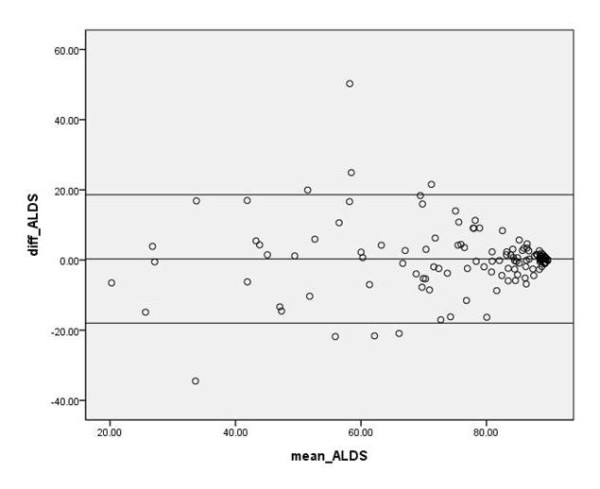
**Bland-Altman analysis of the Academic Medical Center Linear Disability Score**. Bland-Altman analysis of the Academic Medical Center Linear Disability Score (ALDS) for all patients (*n *= 140).

An excellent significant intra-class correlation was found for the ALDS (0.857) and a good intra-class correlation for the PCS (0.798) and the MCS (0.679) of the SF-12 between patients and their proxy (all *P *< 0.001; Table 2). The ALDS was almost the same between patients and proxies (0.1) and for the PCS (-0.1) and the MCS (-0.6) (Table 3). When comparing the proxies regarding gender (*P *= 0.251), relationship with the patient (spouse vs. child; *P *= 0.889) and age (< 65 years vs. ≥65 years; *P *= 0.904), we found no significant differences. Bland-Altman analysis showed that the patients and proxies tend to agree most strongly when the ALDS exceeds 80 (Table 4 and Figure 2).

**Table 2:** Intraclass correlation coefficients for the ALDS, PCS and MCS (SF-12) between patients and their proxies

	All patients (*n *= 140)			All ICU patients (*n *= 70)			ICU scheduled admissions (*n *= 14)			ICU emergency admissions (*n *= 56)			All emergency CCU patients (*n *= 70)		
	**ICC**	**95% CI**	***P *value**	**ICC**	**95% CI**	***P *value**	**ICC**	**95% CI**	***P *value**	**ICC**	**95% CI**	***P *value**	ICC	**95% CI**	***P *value**

ALDS	0.857	0.806 to 0.896	< 0.001	0.861	0.786 to 0.911	< 0.001	0.755	0.410 to 0.913	0.001	0.889	0.817 to 0.934	< 0.001	0.855	0.776 to 0.907	< 0.001
SF-12															
PCS	0.798	0.728 to 0.851	< 0.001	0.789	0.681 to 0.863	< 0.001	0.758	0.369 to 0.920	0.001	0.785	0.661 to 0.879	< 0.001	0.807	0.706 to 0.876	< 0.001
MCS	0.679	0.579 to 0.759	< 0.001	0.612	0.443 to 0.739	< 0.001	0.587	0.097 to 0.852	0.014	0.630	0.445 to 0.764	< 0.001	0.740	0.610 to 0.830	< 0.001

Summary of intraclass correlation coefficients (ICCs) of the Academic Medical Center Linear Disability Score (ALDS) and the Short Form 12 (SF-12) physical component score (PCS) and mental component score (MCS) summary scores between patients and their proxies for all patients, for all ICU patients, for ICU scheduled admissions, for ICU emergency admissions and for coronary care unit (CCU) patients separately. CI, confidence interval.

**Table 3:** Mean ALDS, PCS and MSC for patients and proxies

	All patients (*n *= 140)		All ICU patients (*n *= 70)		ICU scheduled admissions (*n *= 14)		ICU emergency admissions (*n *= 56)		All emergency CCU patients (*n *= 70)	
	**Patient**	**Proxy**	**Patient**	**Proxy**	**Patient**	**Proxy**	**Patient**	**Proxy**	**Patient**	**Proxy**

ALDS	76.4 ± 17.4	76.3 ± 17.2	75.7 ± 18.4	75.6 ± 18.4	76.4 ± 17.6	76.9 ± 20.7	75.3 ± 18.8	75.1 ± 18.1	77.0 ± 16.4	77.1 ± 16.0
PCS	36.0 ± 12.5	36.1 ± 12.8	35.7 ± 12.2	35.7 ± 11.7	42.5 ± 9.1	41.6 ± 11.1	34.2 ± 33.2	34.4 ± 11.5	36.3 ± 12.9	36.7 ± 14.0
MCS	43.4 ± 12.2	44.0 ± 13.1	44.2 ± 11.9	44.9 ± 12.5	41.8 ± 15.8	46.3 ± 16.2	44.8 ± 11.0	44.7 ± 11.6	42.4 ± 12.6	43.1 ± 13.7

Data presented as the mean ± standard deviation. Mean Academic Medical Center Linear Disability Score (ALDS), physical component score (PCS), mental component score (MSC) for patients and proxies for all patients, for all ICU patients, for ICU scheduled admissions, for ICU emergency admissions and for coronary care unit (CCU) patients separately.

**Table 4:** Bland-Altman analyses of the ALDS for patients and proxies

	All patients (*n *= 140)	All ICU patients (*n *= 70)	ICU scheduled admissions (*n *= 14)	ICU emergency admissions (*n *= 56)	All emergency CCU patients (*n *= 70)
Mean difference	0.1	0.1	-0.5	0.2	-0.1
Limits of agreement	-18.6 to 18.5	-18.9 to 19.0	-33.6 to 33.1	-13.4 to 13.6	-18.2 to 18.3

Bland-Altman analyses of the Academic Medical Center Linear Disability Score (ALDS) for patients and proxies for all patients, for all ICU patients, for ICU scheduled admissions, for ICU emergency admissions and for coronary care unit (CCU) patients separately.

#### All ICU patients

In the group of all ICU patients, an excellent intra-class correlation was found between patients and their proxy for the ALDS (0.861) and a good intra-class correlation for the PCS (0.789) and the MCS (0.612) (all *P *< 0.001; Table 2). The mean ALDS was almost the same between proxies and patients, and also the PCS (Table 3). The mean MCS showed a minor difference of -0.7 (Table 3). When comparing the proxies regarding gender (*P *= 0.770), relationship with the patient (spouse vs. child; *P *= 0.654) and age (< 65 years vs. ≥65 years; *P *= 0.744), we found no significant differences.

#### Scheduled ICU admissions versus emergency ICU admissions

In both scheduled and emergency admissions, a significant excellent-good intra-class correlation was found between patients and their proxy for the ALDS, although the lowest correlation was found for the ICU scheduled admissions (0.755) compared with the ICU emergency admissions (0.889). For the PCS, the intra-class correlation was somewhat higher in the ICU emergency admissions (0.785) compared with the ICU scheduled admissions (0.758). The intra-class correlation of the MCS, however, was lower in the ICU scheduled admissions (0.587) compared with the ICU emergency admissions (0.630) (Table 2). The mean ALDS was almost the same regarding the ICU scheduled admission between patient and proxy (-0.5) and for the emergency admissions (0.2) (Table 3). When comparing the proxies in the scheduled admissions regarding gender (*P *= 0. 280), relationship with the patient (spouse vs. child; *P *= 0.192) and age (< 65 years vs. ≥65 years; *P *= 0.464), and in the emergency admissions regarding gender (*P *= 0.377), relationship with the patient (spouse vs. child; *P *= 0.864) and age (< 65 years vs. ≥65 years; *P *= 0.937), we found no significant differences.

#### All emergency coronary care unit patients

The highest intra-class correlation was found between patients and their proxies in the group of CCU patients for the ALDS (0.855; *P *< 0.001) and the PCS (0.807; *P *< 0.001). The intra-class correlation of the MCS (0.740; *P *< 0.001) was also good between patients and proxies (Table 2). Interestingly, in all CCU patients the proxies overestimated the functional and mental status (PCS and MCS). The mean ALDS score was almost the same between patient and proxy (-0.1) (Table 3). When comparing the proxies regarding gender (*P *= 0.053), relationship with the patient (spouse vs. child; *P *= 0.516) and age (< 65 years vs. ≥65 years; *P *= 0.904), we found no significant differences.

## Discussion

The present study shows that relatives in close contact with the patient can adequately reflect the patient's functional status on admission to the ICU by use of the ALDS item bank and the SF-12. When comparing the physical disability score of the ALDS with the PCS of the SF-12, the highest intra-class correlation between patients and their proxies was found using the ALDS item bank. The ALDS could therefore be a useful alternative for the PCS of the SF-12.

To our knowledge the ALDS item bank has not yet been used in critically ill patients. In earlier studies by our group we used the SF-36 to measure HRQOL [3,21-23]. A major advantage of the ALDS item bank for measuring the functional status compared with a sum-score-based questionnaire like the SF-36 is the possibility to arrange both the item difficulty and the patient's ability on a single hierarchical linear scale [4]. Items can be selected for individual patients using small sets of items tailored to the disability level of the patients [14]. Another advantage of the ALDS item bank is its simplicity to use for both patients and their relatives directly after ICU or CCU admission.

The results of the present study are in line with an earlier study by our group, where we used proxies to assess the patient's HRQOL, and a significant correlation was found between the patient's HRQOL and the assessment made by the proxies [3]. In this study the proxies adequately reflect the patient's quality of life (especially the physical functioning) on admission to the ICU when the SF-36 questionnaire was used [3]. Rogers and colleagues [2] and Crispin and colleagues [24] showed that proxies reliably assessed the patient's HRQOL at time of discharge from the ICU. However, in specific dimensions - especially in the area of mental well-being - agreement between patients and proxies was moderate. Other studies have reported similar results [1,2,25,26].

Relatives may be more appropriate in their assessment of the patient's physical health rather than their mental health estimation [1-3,27,28]. These results are also in line with our current study where the correlation between proxies and patients was lower in the MCS of the SF-12 compared with the PCS in all patients and the highest in the functional disability score (ALDS). Additionally, proxies may underreport good quality of life and overestimate poor quality of life [29].

Scales and colleagues reported poor agreement between proxies and patients in a cohort following ARDS survivors [30]; however, patients were asked to estimate their pre-admission HRQOL 3 months following ICU discharge while proxies rated the HRQOL around the time of ICU admission. This delay prior to patients completing the pre-admission HRQOL will probably influence their pre-admission responses to some extent [30]. Furthermore, different from our study population, these proxy estimations were studied in a population with high disease severity [30]. Interestingly, Capuzzo and colleagues found in their study that patients with planned ICU admission had a good memory of their health status before ICU admission after 3 months, suggesting that patients could be asked later to recall their pre-admission health status. Most ICU patients are acute admissions, however, and consequently the results of this study do not demonstrate that the findings reported are of value for patients with acute admissions [31].

Additionally, the gender of the proxy and the amount of contact between the proxy and the patient could influence their capacity to answer questions about the patient's HRQOL before ICU admission [32]. Holman and colleagues showed that some of the ALDS items may have different measurement characteristics for males and females and for younger and older respondents [32]. In our study, however, we found no significant differences between male and female or age regarding the ALDS. Furthermore, when comparing the relationship of the proxies regarding the patients we found no significant differences when the ALDS item bank was used. Existing research demonstrates also that the nature of the patient-proxy relationship does not affect patient-proxy agreement for HRQOL before ICU admission [1,2,28,30]. However, the group of patients in our study was possibly too small to perform this analysis satisfactorily. Proxies can either report from the patient's perspective or they can report from their own perspective. Mc Phail and colleagues found in their study that it was important to give clear instructions regarding the perspective from which the proxies should provide their response [33]. In keeping with this recommendation, proxies in the present study were provided with standard instructions in which they were asked to view the patient's perspective. This prospective was selected because most of our proxies knew the patient very well and they were reporting on behalf of patients with sound cognition.

Finally, we speculated that the acute illness and emergency admission could bias the proxies in the adequacy to assess the patient's functional status. In an earlier study of our group using the SF-36 we found no differences between elective and emergency ICU admissions [3]. This is in line with our current data using the ALDS, which showed no differences between elective and emergency ICU admission.

Several limitations of our study should be mentioned. Firstly, some investigators have raised concerns about proxy estimations of HRQOL in populations with high disease severity [30]. The same study suggested that predictions of poor ICU outcome may be exaggerated if proxies underestimate HRQOL [30]. In contrast to the situation in our previous validation study as well as in the current study, however, where patients and their proxies were interviewed within 72 hours of ICU admission, those investigators interviewed patients 3 months after ICU discharge and interviewed their proxies at study entry. This difference makes it entirely possible that survivors of critical illness may overestimate pre-admission HRQOL. Second, the presence of delirium or cognitive dysfunction at the time of assessment could have influenced the response, although we made an effort to identify and exclude those patients by performing delirium screenings at least twice a day. Third, we included both ICU and CCU patients in our study because the number of patients on the ICU we could ask to fill out the questionnaire was limited due to sedation or mechanical ventilation. We only included acutely admitted CCU patients and think that the severity of disease was comparable with the ICU admissions. Fourth, unfortunately only patients who were conscious and well enough to complete the ALDS item bank could be included in the study. This limitation makes it difficult to generalise the results of our study to all ICU patients. Fifth, the high level of agreement observed in this study could be a potential contributor to the high levels of agreement between patient and proxy. Finally, these data are from a single centre in the Netherlands. Results may differ in other settings and parts of the world.

## Conclusions

Relatives in close contact with the critically ill patient can adequately reflect the patient's functional status on admission to the ICU and CCU by use of the ALDS item bank and the SF-12. When comparing the physical disability score of the ALDS with the PCS of the SF-12, the highest intra-class correlation between patients and their proxies was found using the ALDS item bank. The ALDS could therefore be a useful alternative for the PCS of the SF-12.

## Key messages

• Close relatives of critically ill patients can reflect the patient's disability on ICU and CCU admission when using the ALDS.

• Assessment of the functional health on ICU admission is important to judge the biological reserve of a patient in relation to final outcome.

• The ALDS item bank could be a useful alternative for the PCS of SF-12.

• The advantage of the ALDS item bank is its simplicity of use.

• The ALDS item bank has not yet been used in critically ill patients.

## Abbreviations

ALDS: Academic Medical Center Linear Disability Score; CCU: coronary care unit; HRQOL: health-related quality of life; MCS: mental component score; PCS: physical component score; SF-12: 12-item Short Form Medical Outcomes Study; SF-36: 36-item Short Form Medical Outcomes Study.

## Competing interests

The authors declare that they have no competing interests.

## Authors' contributions

JGMH performed the study, contributed to its design, analysed and interpreted the data, and drafted the article. MGWD contributed to the interpretation and the analysis of the data, and revised the manuscript for important intellectual content. AH contributed to the collection of the data. RB conceived of the study, and revised the manuscript for important intellectual content. LvdB contributed to the collection of the data. PES conceived of the study, contributed to data analysis, its design and the interpretation of the data, and revised the manuscript for important intellectual content. JHR conceived of the study, contributed to its design and the interpretation of the data, and revised the manuscript for important intellectual content. All authors contributed substantially to the manuscript. All authors approved the final version submitted for publication.

## Supplementary Material

Additional file 1 **Academic Medical Center Linear Disability Score item bank**. File containing items from the Academic Medical Center Linear Disability Score item bank. Click here for file
