# Supplementary material for: The Academic Medical Center Linear Disability Score for evaluation of physical reserve on admission to the ICU: can we query the relatives?
Source: Crit Care. 2011 Sep 14;15(5):R212. doi: 10.1186/cc10447 (PMC3334756; doi:10.1186/cc10447)
Supplement: Additional file 1 — Academic Medical Center Linear Disability Score item bank. File containing items from the Academic Medical Center Linear Disability Score item bank. [file cc10447-S1.DOC]

| **Academic Medical Center Linear Disability Score (ALDS) item bank** | | | |  |
| --- | --- | --- | --- | --- |
|  |  |  |  |  |
| **Item** | **Item description** | **Item difficulty** | **Discrimination** | **Linear** |
|  |  | **parameter** | **parameter** | **transformed** |
|  | **Are you able to…** |  |  | ALDS |
| 1 | ...ride a bike for at least 2 hours? | -3.057 | 2.450 | 89 |
| 2 | …vacuum a flight of stairs? | -2.653 | 3.231 | 87 |
| 3 | …carry a bag of shopping upstairs? | -2.140 | 2.702 | 85 |
| 4 | …clean a bathroom? | -1.959 | 3.071 | 84 |
| 5 | …vacuum a room and move light furniture? | -1.879 | 2.455 | 84 |
| 6 | …fetch groceries for 3–4 days? | -1.633 | 2.439 | 82 |
| 7 | …go for a walk in the woods? | -1.504 | 2.562 | 81 |
| 8 | …travel by local bus or tram? | -1.230 | 2.864 | 78 |
| 9 | …walk for more than 15 minutes? | -0.818 | 2.131 | 74 |
| 10 | …carry a tray? | -0.808 | 1.618 | 74 |
| 11 | …walk up a hill or high bridge? | -0.781 | 1.993 | 73 |
| 12 | …go shopping for clothes? | -0.723 | 3.401 | 73 |
| 13 | …cut your toenails? | -0.655 | 1.626 | 72 |
| 14 | …fill in an official form? | -0.614 | 1.028 | 71 |
| 15 | …go to a party? | -0.560 | 1.407 | 70 |
| 16 | …stand for 10 minutes? | -0.525 | 1.834 | 70 |
| 17 | …go to a restaurant? | -0.481 | 1.975 | 69 |
| 18 | …sweep the floor? | -0.450 | 2.872 | 69 |
| 19 | …hang and take in a load of washing? | -0.445 | 2.257 | 69 |
| 20 | …vacuum without moving any furniture? | -0.347 | 2.470 | 67 |
| 21 | …move a bed or table? | -0.304 | 1.342 | 66 |
| 22 | …use a washing machine? | -0.234 | 2.072 | 65 |
| 23 | …reach into a high cupboard? | -0.234 | 1.525 | 65 |
| 24 | …walk up a flight of stairs? | -0.192 | 2.190 | 65 |
| 25 | …go to the bank or post office? | -0.130 | 3.119 | 64 |
| 26 | …walk down a flight of stairs? | -0.020 | 2.620 | 62 |
| 27 | …go to the general practitioner? | 0.020 | 3.289 | 61 |
| 28 | …go for a short walk (15 min)? | 0.071 | 2.059 | 60 |
| 29 | …use a dustpan and brush? | 0.083 | 2.503 | 60 |
| 30 | …write a letter? | 0.175 | 0.862 | 58 |
| 31 | …change the sheets on a bed? | 0.209 | 1.560 | 58 |
| 32 | …cross the road? | 0.224 | 2.906 | 58 |
| 33 | …open and close a window? | 0.240 | 1.417 | 58 |
| 34 | …fetch a few things from the shop? | 0.291 | 2.529 | 56 |
| 35 | …polish shoes? | 0.342 | 1.899 | 56 |
| 36 | …have a shower and wash your hair? | 0.657 | 1.950 | 50 |
| 37 | …fold up the washing? | 0.698 | 1.595 | 50 |
| 38 | …dust? | 0.702 | 2.391 | 50 |
| 39 | …put on/take off lace-up shoes? | 0.759 | 1.584 | 49 |
| 40 | …clean a toilet? | 0.779 | 2.102 | 48 |
| 41 | …make a bed? | 0.842 | 1.732 | 47 |
| 42 | …cut your fingernails? | 0.901 | 1.519 | 46 |
| 43 | …reach under a table? | 0.918 | 1.438 | 46 |
| 44 | …heat tinned food? | 0.922 | 2.572 | 46 |
| 45 | …make eggs or beans on toast? | 1.022 | 3.083 | 44 |
| 46 | …reach into a low cupboard? | 1.092 | 1.513 | 43 |
| 47 | …move between 2 low chairs? | 1.144 | 1.381 | 42 |
| 48 | …pick something up from the floor? | 1.151 | 2.019 | 42 |
| 49 | …clean a bathroom sink? | 1.180 | 2.783 | 42 |
| 50 | …put the washing up away? | 1.263 | 2.001 | 40 |
| 51 | …read a newspaper? | 1.278 | 0.902 | 40 |
| 52 | …get in and out of a car? | 1.339 | 2.174 | 39 |
| 53 | …make porridge? | 1.369 | 2.441 | 39 |
| 54 | …clear the table after a meal? | 1.471 | 2.555 | 37 |
| 55 | …peel and core an apple? | 1.498 | 1.200 | 37 |
| 56 | …prepare breakfast or lunch? | 1.517 | 2.273 | 36 |
| 57 | …clean the kitchen surfaces? | 1.765 | 2.955 | 32 |
| 58 | …put a chair up to the table? | 1.777 | 2.060 | 32 |
| 59 | …eat a meal at the table? | 1.788 | 1.352 | 32 |
| 60 | …wash up? | 1.863 | 2.244 | 31 |
| 61 | …put on/take off socks and slip on shoes? | 1.930 | 1.899 | 30 |
| 62 | …sit up (from lying) in bed? | 1.948 | 1.248 | 30 |
| 63 | …get a book off the shelf? | 2.106 | 1.672 | 28 |
| 64 | …answer the telephone? | 2.148 | 1.156 | 27 |
| 65 | …hang clothes up in a cupboard? | 2.192 | 2.645 | 27 |
| 66 | …make a bowl of cereal? | 2.280 | 2.292 | 25 |
| 67 | …make coffee or tea? | 2.348 | 2.316 | 25 |
| 68 | …put long trousers on? | 2.376 | 2.744 | 24 |
| 69 | …sit on the edge of a bed from lying down? | 2.674 | 1.452 | 21 |
| 70 | …move between 2 dining chairs? | 2.722 | 2.353 | 20 |
| 71 | …wash and dry your lower body? | 2.777 | 3.027 | 20 |
| 72 | …put on/take off a coat? | 2.859 | 2.392 | 19 |
| 73 | …wash/dry your face and hands? | 2.969 | 2.067 | 18 |
| 74 | …get out of bed into a chair? | 2.987 | 2.261 | 18 |
| 75 | …go to the toilet? | 3.077 | 2.954 | 17 |
| 76 | …wash your lower body (at sink)? | 3.235 | 3.140 | 15 |
| 77 | …put on and take off a T-shirt? | 3.494 | 2.690 | 11 |
